# Interleukin-24 enhancing antitumor activity of chimeric oncolytic adenovirus for treating acute promyelocytic leukemia cell

**DOI:** 10.1097/MD.0000000000015875

**Published:** 2019-05-31

**Authors:** Li Liu, Jiabin Ma, Lanyi Qin, Xiaogang Shi, Hongqiang Si, Yahui Wei

**Affiliations:** a Key Laboratory of Resource Biology and Biotechnology in Western China, Department of Life Science, Northwest University, Xi’an, Shannxi; b School of Life Sciences, Zhejiang Sci-Tech University, Hangzhou, Zhejiang, P.R. China.

**Keywords:** acute promyelocytic leukemia, cancer therapy, chimeric oncolytic adenovirus, interleukin-24

## Abstract

**Background:**

Acute promyelocytic leukaemia (APL) is a clonal disease arising by hematopoietic stem cell (HSC), which characterized by inappropriate proliferation/differentiation or survival of immature myeloid progenitors. Oncolytic adenoviruses have been under widespread investigation as anticancer agents. Recently, our data suggested that tumor cells were cured by AdCN205-IL-24, an adenovirus serotype 5-based conditionally replicating adenovirus expressing IL-24 after infection.

**Methods:**

In this study, we created a novel fiber chimeric oncolytic adenovirus AdCN306-IL-24 that has Ad11 tropism and approved CAR (coxsackie adenovirus receptor, CAR)-independent cell entry, which could allow development of selective cytopathic effects (CPE) in APL cells in vitro.

**Results:**

Formidable cytotoxic effect was specifically implemented in APL cells after infection with AdCN306-IL-24. The expression of IL-24 was up-regulated upon treated with accepted tumors. And the vector also induced superior cytolytic effects activity in APL cells by activation of programmed cell death.

**Conclusions:**

Taken together, our data suggested that chimeric oncolytic adenovirus AdCN306-IL-24 could express *IL-24* gene, representing a potential therapeutics for acute promyelocytic leukemia.

## 1 Introduction

Acute promyelocytic leukemia (APL) is a subtype of acute myeloid leukemia (AML), which links to a clonal disorder of hematopoietic stem cells.^[1]^ As the global prediction for this disease remains particularly poor, major efforts have been made to both characterize the deregulated signal transduction pathways in APL cells and to develop targeted therapies against these aberrant signals.^[2,3]^ APL may take place through a variety of molecular genetic alterations affecting activation of signal transduction in different signaling pathways.^[4]^ The mutation signaling proteins are potential molecule marker supporting APL treatment; nevertheless, the development of APL therapies through these signal transduction pathways that overcome enhanced proliferation and survival to hematopoietic cells remain a major challenge.^[5]^ A major number of mutation proteins in signaling pathway, such as PML-RARα fusion, neuroblastoma RAS viral oncogene homolog mutation, have been identified in APL.^[4]^ For these unified approaches, it may represent a more widely applicable treatment strategy in APL. Efforts have been made clinically on the therapeutic targets.^[6,7]^ Researchers lead to a treatment that allows an increase in the curative ratio. At present, cancer gene therapy strategies may provide new hope for expanding APL patients’ treatment regimens.

Gene therapy is as an effective way for regulating biological features in disease systems or curing cancers. Gene therapy has been proved to be a promising approach against cancer. Oncolytic viruses (OVs) not only express therapeutic anti-oncogenes in tumor cells but also can be medicine for destructing the tumor directly. However, the molecular mechanism by which adenoviruses induce tumor cell death remains unclear.^[8–10]^ One common strategy used to devise an oncolytic adenovirus is reliance on selective expression of the viral E1 gene in cancer cell that is mediated by tumor specific transcriptional elements, which are essential for viral replication.^[11,12]^ Because telomerase activity is present in nearly 80% of malignancies, particular attention has concentrated on the upstream regulatory sequence of human telomerase reverse transcriptase (hTERT) as a cancer-specific promoter.^[13]^ Promoter of hTERT has been widely used in cancer gene therapy.^[14]^ A key promoter of hTERT has ample activity in tumor cells and contains different binding sites for ubiquitous transcription factor Sp1 and one E-box at positions where it could bind repressor Mad or activator c-myc. Furthermore, research has illustrated that 3 extra E-boxes in the core promoter led to more firm selectivity for cancer cells. Therefore, the modified or nonmodified hTERT promoter has been applied to cancer-specific replicating adenoviruses.^[15,16]^ Another way to activate adenovirus replication in tumor cells is modification of E1A protein. Moreover, CR2 domain of E1A protein of adenovirus could bind to RB (retinoblastoma) protein, and then the proteins related to RB protein could regulate transcription factors E2F family members and induce stable cells to enter into S-phase. Because RB is dysfunctional in tumor cells, a deletion of CR2 domain allows adenoviruses to replicate specifically in tumor cells.^[17,18]^ The human adenovirus serotype11 (Ad11), different from Ad5 by a fiber, secretes complement regulatory proteins such as CD46 to the plasma. Ad11 may be another choice for leukemia therapy.^[19,20]^ Previously, our work suggested the AdCN205 system for cancer gene therapy tools wherein the intro-promoter, which controls the initiation of *E3* gene of adenovirus, was implemented for controlling expression of therapeutic gene.^[21–25]^ At present, we constructed the AdCN306-11 system, which could selectively replicate in APL cells to exhibit outstanding cytolytic effects activity.

Amelanoma differentiation associated (mda) gene, that is, *mda-7*, is currently listed as a member of the IL-10 cytokine family and is named IL-24.^[26]^ IL-24 is a cytokine with, among cytokines, the most potent anti-tumoral activity. Previous study demonstrated that overexpression of IL-24 containing IL-24 inhibits cell proliferation and induces cell death in a variety of tumor cells (melanoma, cervix, lung, colon, breast, prostate, and human hepatocellular carcinoma), although having no effect on normal cells.^[27–30]^ In addition, IL-24 induces apoptosis, with anti-tumor properties, including cell invasion and metastasis, direct bystander cytotoxicity, anti-angiogenic effects, chemotherapy, ability to make cancer cells sensitive to the therapeutic approaches of radiation, and therapeutic antibodies, been recognized. These results suggested that *IL-24* is an ideal cancer suppressor gene potentially suitable for anti-tumor therapy.^[31,32]^ Our previous work has shown that IL-24 plays strong anti-tumor activity by induction apoptosis of tumor, suggesting inhibition of angiogenesis and enhancement of immune responses.

## 2 Materials and methods

### 2.1 Cell cultures

Human APL cell (HL-60), human liver cell L02, and human embryonic kidney cell HEK293 were purchased from the cell bank of type culture collection of Chinese Academy of Sciences. HL-60 was cultured in RPMI 1640 medium and, L02 and HEK293 were cultured in Dulbecco's modified eagle medium. All mediums were supplemented with 10% fetal bovine serum, 50 U/mL penicillin, and 50 mg/mL streptomycin. Cell culture condition is humidified incubator (37°C) with 5% CO_2_.

### 2.2 Plasmids clone of the chimeric oncolytic adenoviral

The plasmids, containing pCN306-IL-24 and pCN306-EGFP, were obtained according to the protocols. The pCN103 plasmids were constructed previously and reserved in our laboratory. pCN306-IL-24 and pCN306-EGFP plasmids and pCN103 plasmid harboring the oncolytic adenoviral backbone were transducted into BJ5183 strain from *Escherichia coli* to produce pAdCN306-EGFP and pAdCN306-IL-24 in a homologous recombination dependent manner, respectively. Adenoviruses were harvested in HEK293 cells after transfection with endonuclease Pac I digested pAdCN306-EGFP and pAdCN306-IL-24 for 48 hours and recombinant AdCN306-EGFP and AdCN306-IL-24 were obtained.^[33]^

### 2.3 Acquisition of adenovirus

The chimeric oncolytic adenoviruses (AdCN306-EGFP and AdCN306-IL-24) were obtained using standard homologous recombination techniques. The plasmids pCN306-EGFP and pCN306-IL-24 were digested by endonuclease PacI and then the plasmid fragment was transferred into HEK293 cells with the help of Effectene Transfection Reagent (Qiagen, Hilden, Germany); supernatant was harvested at 48 hours later. The resultant recombinant adenovirus was isolated using single plaque and expanded in HEK293. For large-scale purification, viruses were obtained by ultracentrifugation with cesium chloride. The virus titers were measured by a plaque assay in HEK293.

### 2.4 Measurement of virus progeny

To test the viral replication capacity, HL-60 or L02 cells were infected with different adenoviruses at a multiplicity of infection (MOI) of 10. After infection for 48 hours, the cells were obtained and lysed by 3-time cycles of freeze thawing. The outcome was titrated with 50% infectious dose assay in HEK293 cell, and the data were converted into PFU per cell.

### 2.5 Cytotoxicity assay and adenovirus infection

All cells were planted into 96-well plates with 1 × 10^4^ cells per well. Cells that reached subconfluence were infected with AdCN306-EGFP, AdCN306-IL24, AdCN205-EGFP, AdCN205-IL-24, or Ad-wt at MOI of 10. To determine the adenovirus-infected cellular toxicity, the MTT assay was performed as previously described.^[34]^ Absorbance of each well was detected at 595 nm with microplate reader (Bio-Rad, Hercules, CA).

### 2.6 In vitro transduction assay

HL-60 cells were seeded (3 × 10^5^ per-well) 1 day before infection. Then, viruses were infected in 1-mL culture medium. Cells were infected with adenoviruses for 1 hour and then incubated at 37°C. GFP-positive cells were counted using flow cytometry and microscope.

### 2.7 Apoptotic cell staining

The HL-60 cells were grown on glass cover slips in each 6-well plate at a density of 4 × 10^5^ per well. After 12 hours, cells were infected with AdCN306-IL-24, AdCN306-EGFP, AdCN205-EGFP, AdCN205-IL-24, or Ad-wt (MOI of 10). After infection for 48 hours, culture medium was replaced with phosphate-buffered saline (PBS), and then cells were incubated with Hoechst 33342 at a concentration of 25 μg/mL. The programmed death cells were detected by fluorescent microscopy.

### 2.8 Flow cytometric analysis

To determine transduction efficiency of adenovirus, HL-60 cells were seeded into 6-well plates and incubated with adenovirus, AdCN205-EGFP, AdCN306-EGFP, or Ad-wt, with a MOI of 10 for 48 hours, and finally detected with flow cytometry,

### 2.9 Western blotting

For chimeric oncolytic adenovirus characterization, HL-60 cells were planted into 6-well plates and then infected with all adenoviruses (MOI of 10) for 2 days. To evaluate the expression of related proteins, Western blotting was carried out. Briefly, total proteins were lysed with lysis buffer for Western blot or immune-precipitation (IP). Concentration of proteins was quantitated using BCA Protein Assay Kit (Beyotime, Haimen, China). The proteins were separated on 10%SDS-PAGE gel and then transferred to polyvinylidene difluoride membrane (Millipore, Bedford, MA). Membranes were incubated in nonfat dry milk (5%), followed by primary antibodies overnight, and then secondary antibodies. Finally, membranes were exposed to enhanced ECL agent for quantification using chemilluminescence detection system (Bio-Rad). The following primary antibodies were used. Rabbit monoclonal anti-mda-7, Rabbit monoclonal anti-IL-24, and Rabbit polyclonal anti-cleaved caspase-3 were obtained from Abcam (Cambridge, MA) in UK. Rabbit polyclonal anti-poly-(ADP-ribose) polymerase-1/2 (H-250) and Rabbit monoclonal anti-GAPDH were obtained from Santa Cruz, CA in USA.

### 2.10 Statistical analysis

Experiments were performed in at least 3 independent repeats. Student *t* test was carried out to define the significance. *P* < .05 was considered as statistically significant.

## 3 Results

### 3.1 The construction of the chimeric oncolytic adenoviral vectors

**Figure 1:**
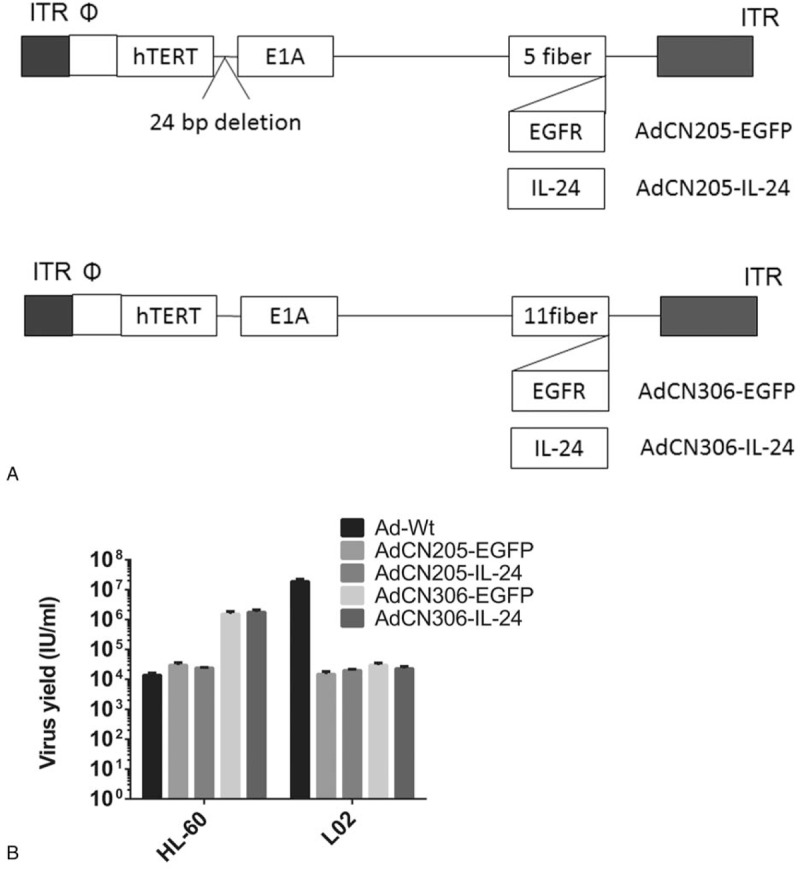
Characterization of chimeric oncolytic adenoviral vectors. (A) Schematic diagram of chimeric oncolytic adenovirus carrying EGFP or IL-24. Diagrams of AdCN205-EGFP, AdCN205-IL-24, AdCN306-EGFP, or AdCN306-IL-24. In the AdCN205 system, E1A promoter was a substitute of hTERT promoter and the deletion of the adenovirus genome 923 to 946 nucleotides make virus replication in malignant tumor cells with abnormal Rb gene function. The fibers on Ad5 were replaced with Ad11. In AdCN205-EGFP, AdCN205-IL-24, AdCN306-EGFP, or AdCN306-IL-24 was substituted by the EGFP and the *hIL-24* therapeutic gene. (B) Tumor selective replication adenoviruses. HL-60 cells and L02 cells were infected with AdCN205-EGFP, AdCN205-IL-24, AdCN306-EGFP, AdCN306-IL-24, or Ad-wt at MOI of 10. After 2 days, supernatants and pellet were harvested separately. Cells were resuspended with PBS and subjected with 3-time freeze-thaw cycles. Virus yield was examined in supernatants and cell lysates. Compared with AdCN205-EGFP or Ad-wt, ^∗^*P* < .01. APL = acute promyelocytic leukemia, IL-24 = interleukin-24, MOI = multiplicity of infection.

Our previous data reported a series set of adenoviruses replicating conditionally in tumor cells with a high level of hTERT protein. Although exogenous constitutive promoters control therapeutic genes in these oncolytic adenoviruses, expression level of these genes may induce unexpected effects, but oncolytic adenoviruses do not replicate in normal cells. For overcoming the limitation, we exploited the AdCN205 system for cancer therapy, and that system could express levels of these genes by regulating E3 endogenous promoter. Our results demonstrated that therapeutic gene was expressed in a secure and predictable pattern. Previous study suggested that Ad11 could secrete the complement regulatory protein CD46 to the plasma. The Ad11 adenovirus is an alternative option for oncotherapy such as leukemia. Next, we constructed 2 vectors AdCN306-EGFP and AdCN306-IL-24 by replacing fibers on Ad5 with Ad11. The schematic diagram of AdCN205-IL24, AdCN205-GFP, AdCN306-IL24, and AdCN306-GFP is shown in Fig. 1A.

### 3.2 Chimeric oncolytic adenoviral vectors selective replication in vitro

APL cells (HL-60) were transduced by *IL-24* gene with high efficiency; we developed a chimeric oncolytic adenoviral vector system with IL-24 (AdCN306-IL-24 and AdCN306-EGFP). To investigate whether selective replication ability of chimeric oncolytic adenoviruses could interfere with transgenes, progeny assay was applied in HL-60 or L02 cells. Cells were incubated with all the adenoviruses at MOI of 10 (Fig. 1B). Among them, AdCN306-EGFP and AdCN306-IL-24 had a similar replication capacity in APL cells, whereas the replication efficiency of these vectors was decreased sharply in L02 cells. These results revealed that IL-24 and EGFP have no effect on selective replication.

**Figure 2:**
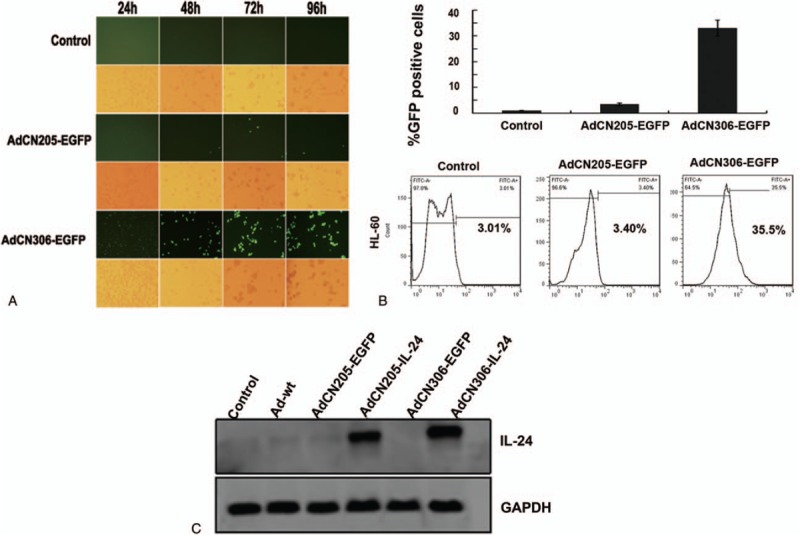
Tumor selective replication of adenoviruses. (A) Images were observed from HL-60 cells that were infected with AdCN306-EGFP, AdCN205-EGFP, or Ad-wt at MOI of 10 (×200). (B) Adenoviruses transduction efficiency. HL-60 cells were infected with AdCN306-EGFP, AdCN205-EGFP, or Ad-wt, at MOI of 10, and then fluorescence was detected using flow cytometry. (C) Protein level of IL-24. HL-60 cells were infected with AdCN306-IL-24, AdCN306-EGFP, AdCN205-EGFP, AdCN205-IL-24, or Ad-wt, at MOI of 10. Total protein after quantification was subjected to Western blotting with antibodies against total IL-24 and GADPH.

Meanwhile, to confirm whether exogenous genes were expressed normally in APL cells, cells were infected with chimeric oncolytic adenoviruses. HL-60 cells were incubated with all the adenoviruses at MOI of 10 (Fig. 2A). In HL-60 cells, AdCN306-EGFP group had an upregulated expression, compared with that of AdCN205-EGFP group under a fluorescence microscope.

In addition, to evaluate the efficiency of recombinant oncolytic adenoviral vectors, HL-60 cells were incubated with all the adenoviruses at MOI of 10, and then cells were analyzed using flow cytometry (Fig. 2B). AdCN306-EGFP had specificity and selectively higher replication ability in HL-60 cells and then Ad-wt or AdCN205-EGFP. To investigate whether IL-24 expressed by exogenous promoter could lead to apoptotic events in APL cells, APL cells were incubated with all the adenoviruses at MOI of 10. Key proteins were assayed by Western blotting (Fig. 2C).

In summary, these data suggested that IL-24 expressing chimeric oncolytic adenoviral vector could infect and replicate in APL cells with a high-level expression of the harboring genes, but not in normal cells.

### 3.3 Cytotoxic effects of chimeric oncolytic adenoviral vectors on acute promyelocytic leukemia in vitro

**Figure 3:**
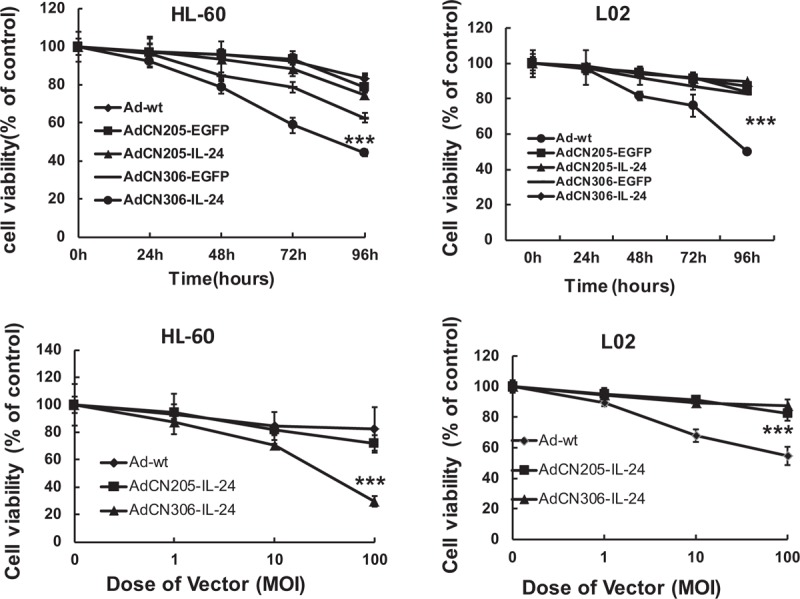
Cytotoxicity of chimeric oncolytic adenoviral infected cells. HL-60 or L02 cells were infected with AdCN306-EGFP, AdCN205-IL-24, AdCN306-IL-24, AdCN205-EGFP, or Ad-wt at MOI of 10. Cell viability was resolved to (3-[4,5-dimethylthiazol-2-yl]-2,5-dipenyltetrazolium bromide) assay at 1, 2, 3, and 4 days after infection. The cell viability was reckoned to cells without viral infection. Data were deviation of 3 independent experiments. ^∗^*P* < .01 compared with groups of AdCN205-EGFP and Ad-wt.

To analyze the anti-tumor capability of the chimeric oncolytic adenoviruses with IL-24, we executed a cytotoxicity assay after infection with adenoviruses (Fig. 3). HL-60 and L02 cells were incubated with the adenoviruses. AdCN205-IL-24, AdCN205-EGFP, or Ad-wt induced a decreased viability of APL cells at nearly equal level in a time-dependent manner, but AdCN306-IL-24 had a more effective cytotoxicity than other viruses for APL cell. Surprisingly, AdCN306-EGFP or AdCN306-IL-24 had no equal effect on cytotoxicity in L02 cells. In addition, we examined increased cytotoxicity in a dose-dependent manner in HL-60 cells (Fig. 3B).^[23]^ These results revealed that AdCN306-IL-24 and AdCN306-EGFP selectively replicate in cancer cells and even AdCN306-IL-24 showed a better efficacy in APL.

### 3.4 Apoptosis in acute promyelocytic leukemia cells after infection with chimeric oncolytic adenoviral

**Figure 4:**
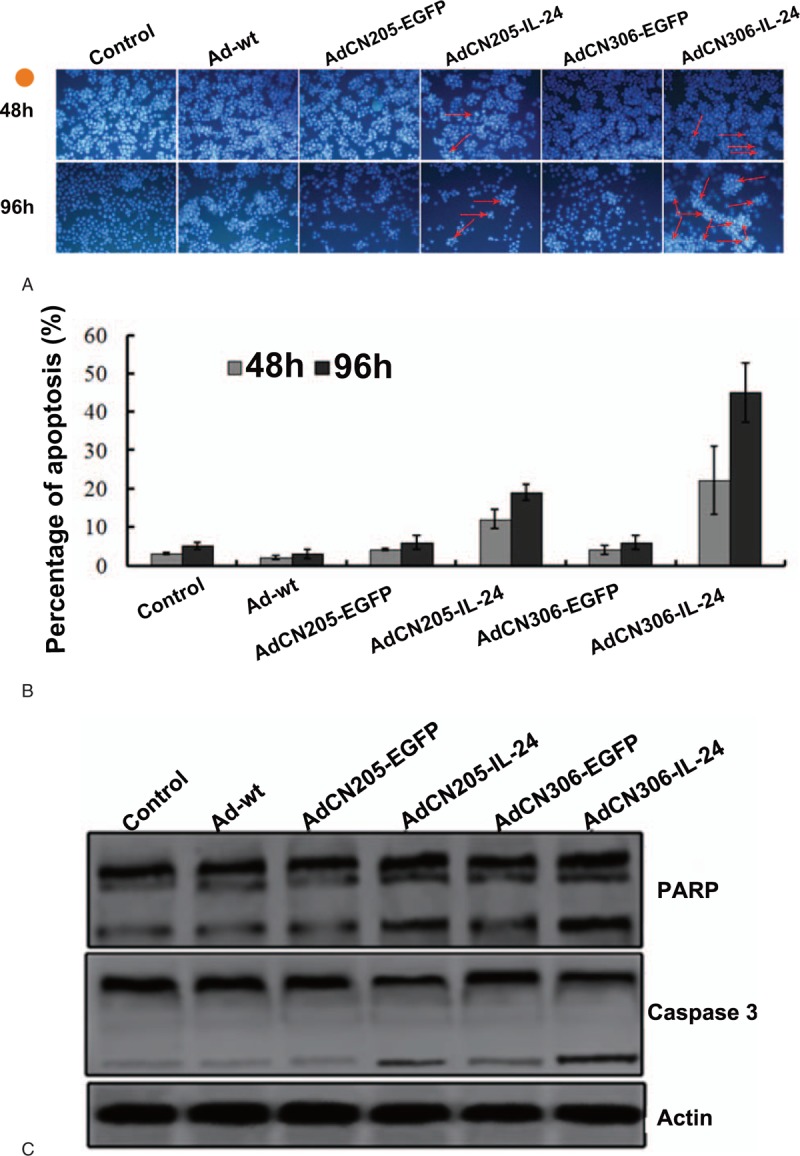
Apoptosis in IL-24 positive APL cells. (A, B) HL-60 cells were infected with AdCN306-EGFP, AdCN205-IL-24, AdCN306-IL-24, AdCN205-EGFP, or Ad-wt at MOI of 10 for 2 or 4 days and then cells were incubated with Hoechst 33342. Arrows indicate positive apoptotic cells (×100). (C) Protein level of cleaved caspase 3 or PARP. HL-60 cells were infected with AdCN205-IL-24, AdCN306-EGFP, AdCN306-IL-24, AdCN205-EGFP, or Ad-wt at a MOI of 10 for 2 days. The quantified proteins were subjected to Western blotting with antibodies.

To investigate whether exogenous expression of IL-24 could induce programmed cell death in APL cells, HL-60 cells were incubated with the adenoviruses. Infection of HL-60 cells with AdCN306-IL-24 caused tremendous morphological changes representing apoptosis (Fig. 4). Our data suggested that apoptotic proportion induced by AdCN306-IL-24 was markedly higher than that induced by other viruses. Also, activation of the caspase-dependent pathway was inspected to account for the apoptosis of APL cell. Expression of critical proteins was involved in apoptosis pathways, which were assessed by Western blotting (Fig. 4C). The PARP and cleaved caspase 3 have shown markedly increased intensity, compared with that infected with AdCN306-IL-24 compared with AdCN205-EGFP, Ad-wt, AdCN306-EGFP, AdCN306-IL-24, or blank control. These data indicated that AdCN306-IL-24 could remarkably induce APL cell apoptosis by activation of caspase-dependent pathway (Fig. 4C).

## 4 Discussion

APL is a bone marrow derived malignancy characterized by a differentiation block at early stage of promyelocytic. The biological basis for APL has been studied in terms of morphology, cytogenetics, responsiveness to hematopoietic growth factors, cell membrane antigen expression, degree of leukemic transformation, and oncogene expression. The APL pathogenesis progress features may be partially attributed to the disruption of transduction of special signal pathways or differentiation of hematopoietic cells. Knowledge acquired from these studies focuses on a large number of components of signaling pathway that confer enhanced proliferation and survival to hematopoietic stem cells in APL. These results offer opportunities to identify other signaling events with a role in APL pathogenesis. Eventually, it may provide a strategy to anti-APL by means of signaling transduction inhibitors.

Studies have revealed a large number of unique features of mda-7/IL-24. IL-24 can be described as a “magic bullet,” including tumor-specific apoptosis induction, potent “bystander” effect, and ability to make cancer cells more sensitive to clinical therapeutic approaches, including radiation, chemical drugs, and immune antibodies. IL-24 is a cytokine and potential powerful therapeutic agent for anti-cancer.^[29,32]^ Previous work of our group has defined that the cytokine, IL-24, had powerful anti-tumor activity, in vitro or in vivo.^[23]^ Here, we explored whether *IL-24* is a potential therapeutic gene for the treatment of APL.

To target APL cells and improve safety in noncancer cells, oncolytic adenovirus harboring therapeutic genes could be one of effectively important measures against APL. The viral system, competently replicating in APL cell, may provide a better choice for treatment. Several studies have demonstrated that oncolytic adenoviruses have the capacity to transfect tumor cells, and display effectiveness and relative safety for anti-tumor.^[10]^

Our previous research indicated that AdCN205 system, which is a double-regulated oncolytic adenoviral system, could be constructed to target hTERT and RB pathway.^[21]^ For the regulation of transgenes, the double-regulated oncolytic adenoviral vector could express the carrying genes limited to cancer cells. The expression of IL-24 could cooperate with oncolytic effects and enhance the effects of adenoviral vectors, in vitro or in vivo. Moreover, we constructed oncolytic adenoviral vectors by replacing the fibers on Ad5 with Ad11.^[35,36]^

Our data illustrated that AdCN306-IL-24 had robust cytotoxic effect on APL cells compared with other vectors (AdCN205-IL-24, AdCN205-EGFP, or Ad-Wt). AdCN205-EGFP, AdCN205-IL-24, and Ad-wt vectors have same cytotoxic effects as APL cells. Meanwhile, AdCN205-EGFP or AdCN205-IL-24 had a cytotoxic effect on normal cells, but AdCN306-IL-24 did not. In addition, we revealed that *IL-24* gene transduced by AdCN306-IL-24 could be significantly expressed in APL cells. These results suggested that our recently constructed chimeric oncolytic adenoviral vector system, AdCN306-IL-24, had a capacity of replication in APL cells and, at the same time, expression of the transduced gene.

These results implied the cytotoxic effects of AdCN306-IL-24 on APL cells induced by programmed cell death intermediated by activation of cleaved caspase 3 and PARP, which is a critical mechanism for induction of caspase-dependent network in apoptosis.^[37]^

## 5 Conclusion

We created a tumor-selective chimeric oncolytic adenovirus system in an efficient way. AdCN306-IL-24 selectively replicate in APL cells and showed anti-tumor activity. Also, the *IL-24* gene in chimeric oncolytic adenovirus induces programmed cell death through caspase-dependent apoptosis manner. The study provides a promising strategy for therapy of APL and IL-24 might also be applied as a useful tool in cancer gene therapy as well. In addition, there were some limitations in the work. Increased MOI may induce reduction of cell viability. The whole effects on viability of cells may be induced by high MOI, bystander effect, or both. However, we could not confirm that which one was the major one. This issue may be the aim of our future work.

## Author contributions

**Conceptualization:** Li Liu, Yahui Wei.

**Investigation:** Lanyi Qin.

**Methodology:** Li Liu.

**Project administration:** Yahui Wei.

**Software:** Jiabing Ma, Xiaogang Shi, Hongqiang Si.
